# Genome-scale comparison and constraint-based metabolic reconstruction of the facultative anaerobic Fe(III)-reducer *Rhodoferax ferrireducens*

**DOI:** 10.1186/1471-2164-10-447

**Published:** 2009-09-22

**Authors:** Carla Risso, Jun Sun, Kai Zhuang, Radhakrishnan Mahadevan, Robert DeBoy, Wael Ismail, Susmita Shrivastava, Heather Huot, Sagar Kothari, Sean Daugherty, Olivia Bui, Christophe H Schilling, Derek R Lovley, Barbara A Methé

**Affiliations:** 1 Department of Microbiology, 203N Morrill Science Center IVN, University of Massachusetts Amherst, Amherst, MA 01003, USA; 2 Genomatica Inc, 10520 Wateridge Circle, San Diego, CA 92121, USA; 3 University of Toronto, 200 College St, Toronto, ON M5S3E5, Canada; 4 J Craig Venter Institute, 9712 Medical Center Drive, Rockville, MD 20850, USA; 5 Institute for Genome Sciences, University of Maryland School of Medicine, Baltimore, MD 21201, USA

## Abstract

**Background:**

*Rhodoferax ferrireducens *is a metabolically versatile, Fe(III)-reducing, subsurface microorganism that is likely to play an important role in the carbon and metal cycles in the subsurface. It also has the unique ability to convert sugars to electricity, oxidizing the sugars to carbon dioxide with quantitative electron transfer to graphite electrodes in microbial fuel cells. In order to expand our limited knowledge about *R. ferrireducens*, the complete genome sequence of this organism was further annotated and then the physiology of *R. ferrireducens *was investigated with a constraint-based, genome-scale *in silico *metabolic model and laboratory studies.

**Results:**

The iterative modeling and experimental approach unveiled exciting, previously unknown physiological features, including an expanded range of substrates that support growth, such as cellobiose and citrate, and provided additional insights into important features such as the stoichiometry of the electron transport chain and the ability to grow via fumarate dismutation. Further analysis explained why *R. ferrireducens *is unable to grow via photosynthesis or fermentation of sugars like other members of this genus and uncovered novel genes for benzoate metabolism. The genome also revealed that *R. ferrireducens *is well-adapted for growth in the subsurface because it appears to be capable of dealing with a number of environmental insults, including heavy metals, aromatic compounds, nutrient limitation and oxidative stress.

**Conclusion:**

This study demonstrates that combining genome-scale modeling with the annotation of a new genome sequence can guide experimental studies and accelerate the understanding of the physiology of under-studied yet environmentally relevant microorganisms.

## Background

*Rhodoferax ferrireducens *is of interest because of its potentially important role in carbon and metal cycling in soils and sediments and its novel ability to convert sugars into electricity [1]. *R. ferrireducens*, which was isolated from subsurface sediments in Oyster Bay, VA, is a facultative anaerobic microorganism in the *Comamonadaceae *family of the *Betaproteobacteria *[2]. It is one of the few known facultative microorganisms that can grow anaerobically by oxidizing organic compounds to carbon dioxide with Fe(III) serving as the electron acceptor. This property, as well as its ability to grow at the low temperatures found in many subsurface environments, suggests that it could contribute to the oxidation of organic matter coupled to the reduction of Fe(III) in many soils and sediments. Microorganisms closely related to *R. ferrireducens *have been detected in a number of subsurface environments [3-7]. The novel ability of *R. ferrireducens *to oxidize sugars to carbon dioxide with quantitative electron transfer to electrodes in microbial fuel cells is of interest because of the possibility of using sugars as a renewable energy source for power production [1,8,9].

*R. ferrireducens *has a number of important physiological characteristics that distinguishes it from other members of the genus *Rhodoferax*. For example, it appears to be unable to grow phototrophically [2], a previous hallmark feature of the genus [10,11]. Furthermore, unlike other *Rhodoferax *species, *R. ferrireducens *cannot grow anaerobically via fructose fermentation. No other *Rhodoferax *species have been shown to grow via anaerobic respiration, whereas *R. ferrireducens *can grow by oxidizing a wide variety of organic electron donors, such as acetate, lactate, propionate, pyruvate, succinate, malate and benzoate, with Fe(III) serving as the electron acceptor [2]. In addition to Fe(III), *R. ferrireducens *can utilize Mn(IV) oxide, fumarate, and nitrate as electron acceptors to support anaerobic growth [2].

The production of linear polyesters in the form of polyhydroxyalkanoates (PHAs) [2] is an interesting characteristic of *R. ferrireducens *with important biotechnological implications. PHAs are typically synthesized in bacteria from sugars or lipids and have industrial interest due to their properties as thermoplastics and elastomers [12].

In order to further elucidate the physiology of *R. ferrireducens*, the publicly available genome sequence  was annotated in more detail and a genome-scale metabolic model was reconstructed using the constraint-based modeling approach [13-15]. Constraint-based modeling couples stoichiometric reconstructions of all known metabolic reactions in the organism with a set of constraints on the fluxes of each of these reactions in the system. This approach unveiled a variety of previously unknown physiological features of *R. ferrireducens *that contributed to a better understanding of its potential role in subsurface environments and converting organic compounds to electricity.

## Results and Discussion

### General features of the genome

**Figure 1:**
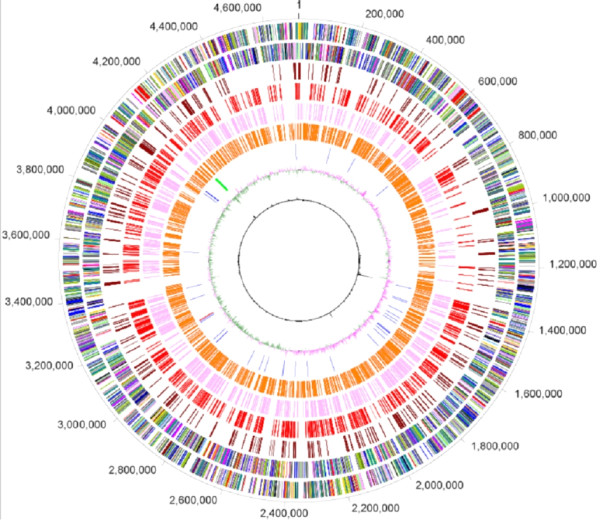
**Graphic representation of the *Rhodoferax ferrireducens *genome**. The first two outer circles represent the positions of genes in the *Rhodoferax *chromosome (Circle 1- plus strand, Circle 2- minus strand). Circles 3-6 represent *Rhodoferax *genes with a bidirectional best match to *Polaromonas *JS666, and they are grouped according to percent identity of each BLAST match. Circle 7 represents rRNA genes (green ticks), tRNA genes (blue ticks) and sRNA genes (red ticks).

**Figure 2:**
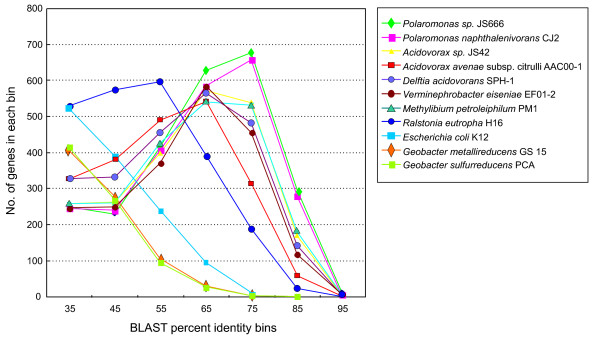
***R. ferrireducens *BLAST matches to fully sequenced genomes**. Curves summarizing BLAST matches for *Rhodoferax *genes sorted by matching organism and binned according to percent identity of each BLAST match. Each curve represents a different matching organism (see color code in key), and only the top 10 fully sequenced genomes are shown. The x-axis coordinate is binned according to the percent identity for a group of BLAST matches, and the y-axis coordinate indicates the number of *Rhodoferax *genes in a particular group.

The *Rhodoferax ferrireducens *genome as sequenced and assembled by the Joint Genome Institute (JGI) [16] consists of a circular chromosome of 4,712,337 base pairs (bp) and a plasmid with 257,447 bp. Manual curation of this sequence predicted a total of 4451 **c**o**d**ing **s**equences (CDSs) from the chromosome and 319 CDSs from the plasmid (Figure 1, Table 1). Of the chromosomally located CDSs, BLAST searches matched 3,830 to a database of Proteobacterial proteins, by selecting for alignments with a 70% length requirement, 35% identity and an e-value less than 1 e^-5^. Analysis of the best BLAST match for each CDS against the database of Proteobacterial proteins established the taxonomic breakdown as follows: *Betaproteobacteria *(3,253 genes), *Gammaproteobacteria *(258), *Alphaproteobacteria *(185), *Delta/Epsilonproteobacteria *(121), and unclassified *Proteobacteria *(13). Beyond examining best BLAST matches, the "BLAST Curves" analysis was also employed. BLAST Curves (Figure 2) visually display the number of genes in each percent identity bin, and each color-coded curve corresponds to matches to a particular genome in the BLAST database. By comparing the profiles of all the curves in the graph, relative rankings of completely sequenced Proteobacterial genomes in the BLAST database can be elucidated for *R. ferrireducens*. For example, a curve having a profile with a peak on the right side of the graph, which indicates high percent identity matches to *R. ferrireducens*, is ranked higher than a curve displaying a peak on the left side of the graph. An important note concerning BLAST Curves is that a database genome with a lower absolute number of matches can have a more significant ranking if it has a high percent identity profile compared to another genome with a higher absolute number of low percent identity matches. As shown in Figure 2, the genomes in the BLAST database, which are most closely related to *R. ferrireducens*, are *Polaromonas *sp. JS666 and *Polaromonas napthtalenivorans *CJ2 species with 2508 and 2417 matches respectively [17-20]. The next several closely ranked organisms include *Acidovorax *sp. JS42 and *Acidovorax avenae *subsp citrulli AAC00-1 (both with 2203 matches), *Delftia acidovorans *SPH-1 (2308 matches), and *Verminephrobacter eiseniae *EF01-2 (2029 matches). Compared to these examples of *Comamonadaceae*, the unclassified Burkholderiales, *Methylibium petroleiphylum *PM1 (2116 matches) and *Ralstonia eutropha *H16 (2303 matches) have a lower profile in the BLAST Curves output. *R. ferrireducens *is also a better match to the *Gammaproteobacterium Escherichia coli *K12 (1259 matches) than to the *Deltaproteobacteria Geobacter metallireducens *GS 15 and *G. sulfurreducens *PCA (825 and 811 matches, respectively).

**Table 1:** General features of the *R. ferrireducens *genome.

	**Chromosome**	**Plasmid**
Size (bp)	4,712,337	257,447
G+C percentage	59.9	54.4
Predicted CDSs	4,451	319
Average size of CDS (bp)	959	714
Percentage coding	91.4	89.6
Number of rRNA operons (16S-23S-5S)	2	0
Number of tRNAs	44	1
Number of CRISPR loci	1	1
Number of conserved hypothetical proteins	669 (14%)	33 (10.3%)
Number of hypothetical proteins	656 (13.8%)	226 (70.8%)

Of the 319 predicted CDSs in the plasmid, BLAST searches matched only 69 to a database of Proteobacterial proteins, using the same criteria used for the chromosome (see above). These include possible conjugation proteins, 3 integrases separated by large spans of hypothetical proteins, a helicase, a DNA methylase, a gene for DNA repair protein RadA, a copy of polymerase DnaN (different from the chromosome copy), at least 6 CRISPR *cas *genes, a DNA ligase, a thymidine kinase, 3 secretion proteins, 2 sensor histidine kinases, and possible type 4 pilin proteins. An unusual finding was a copy of a tRNA-Ile, which is different in sequence from the identical copies in the chromosome but shares the same anticodon.

### *In silico *constraint-based modeling as a tool to gain new insights into the physiology of *R. ferrireducens*

#### Development of the constraint-based *in silico *model

**Figure 3:**
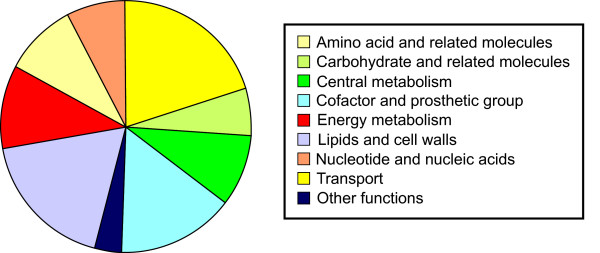
**Functional classification of metabolic reactions in the *R. ferrireducens *model**.

Of the 4770 genes in the *R. ferrireducens *genome used for developing the *in silico *model, 744 genes were included in the reconstructed genome-scale network. The *R. ferrireducens *metabolic model contains 762 reactions and 790 metabolites, including 69 extracellular metabolites (Table 2). A detailed list of genes, reactions, metabolites, and gene-protein-reaction (GPR) associations in the metabolic model are available in Additional file 1. The functional characterization of the 762 reactions in the model is summarized in Figure 3. Among different functional groups, reactions for biosynthesis of amino acids, lipids and cell wall components, cofactors, and nucleic acids are the most abundant, accounting for 65% of all the reactions. Currently, there are 77 reactions associated with transporting metabolites, including redundant transporters for the extracellular metabolites.

**Table 2:** Characteristics of the *R. ferrireducens *genome-scale model.

	***R. ferrireducens***	
**Total Genes**	4770	
Included Genes	744	(15.6%)
Excluded Genes	4026	(84.4%)
**Total Proteins**	653	
**Total Reactions**	762	
Non-gene Reactions	48	(6.3%)
Input/Output Reactions	69	
**Total Metabolites**	790	
Extracellular Metabolites	69	(8.7%)

#### Stoichiometry of the electron transport chain

**Figure 4:**
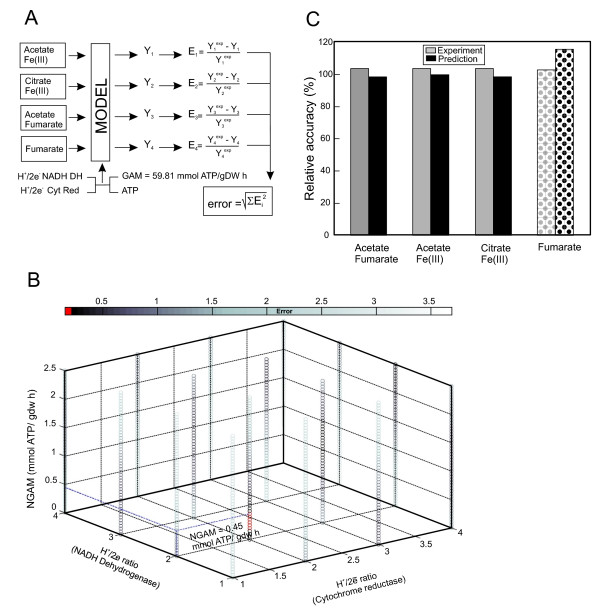
**Determination of energy parameters**. A) Diagram of the algorithm utilized in the determination of proton translocation stoichiometry and NGAM. A range of possible H^+^/2e^- ^values for both NADH dehydrogenase and cytochrome reductase and NGAM values are iterated by the algorithm so that the "error" (E) between predicted and experimentally determined yields (Y) is minimized. The model was constrained with a fixed GAM and growth rates ≤ 0.04 h^-1 ^to match actual conditions. B) Representation of errors between model predictions and experimental data. The lowest errors highlighted in red. C) Comparison between predicted (black bars) and experimental (gray bars) yields. Values are presented as ratios, with experimental yields set at 100%. Yields were obtained from cultures in the following conditions: 10 mM acetate/40 mM fumarate; 56 mM ferric citrate; 20 mM acetate/10 mM Fe(III)NTA. The patterned columns represent the validation data by growth on fumarate as the sole substrate (30 mM). Values are presented as ratios, with experimental yield set at 100%.

The model incorporates two types of maintenance energy parameters: the growth-associated maintenance (GAM), which reflects the energy cost during division (*e.g*. protein polymerization); and the non-growth associated maintenance (NGAM), which reflects the basal energy requirement of the cell regardless of growth. Based on the similarity in biomass composition between *R. ferrireducens *and *E. coli*, the GAM was set at 59.81 mmol ATP/gdw h, the same as the *E. coli *model [21]. Three independent sets of physiological and growth data were used to determine the NGAM and the stoichiometry of the electron transport chain of the genome-scale model of *R. ferrireducens*: 1) acetate and fumarate, 2) acetate and Fe(III) [2] and 3) citrate and Fe(III) (see Methods for growth conditions). In order to determine the most consistent values with experimentally determined growth yields, an optimization algorithm (Figure 4A) iterated the H^+^/2e^- ^ratio of NADH dehydrogenase from 1 to 4, the H^+^/2e^- ^ratio of cytochrome reductase from 1 to 4, and the NGAM from 0 to 2.5 mmol ATP/gdw h. This process identified optimal energy parameters of an H^+^/2e^- ^ratio of 2 for both NADH dehydrogenase and cytochrome reductase, and an NGAM of 0.45 mmol ATP/gdw h (Figure 4B). This set of energy parameters was applied to the metabolic model in simulations to compare between *in silico *predictions and experimentally determined yields. A fourth set of experimental data, obtained from a batch culture grown on fumarate as the electron donor and acceptor, was used to validate the model. The biomass yield of fumarate was calculated by constraining fumarate, succinate, and acetate fluxes to simulate the experimental conditions, and the yields obtained through the simulation closely matched the actual experimental results (Figure 4C). The use of fumarate as electron donor and acceptor by *R. ferrireducens *is further described in the fumarate dismutation section.

It is interesting to point out that the obtained NGAM for the *R. ferrireducens *genome-scale model is similar to that of *G. sulfurreducens*, another acetate-oxidizing Fe(III) reducer [22] often found in the same microbial niche [3-7]. Both *R. ferrireducens *and *G. sulfurreducens in silico *models have an H^+^/2e^- ^ratio of 2 for the NADH dehydrogenase of the electron transfer chains. However, the H^+^/2e^-^ratio for cytochrome reductase is 2 in *R. ferrireducens *as opposed to 1 in *G. sulfurreducens *[22]. Such a difference between the two models implies that the electron transfer chain of *R. ferrireducens *is more efficient than that of *G. sulfurreducens*. The evolution of electron transport chains with different efficiencies suggests that microorganisms could adapt to different lifestyles within the same community. Additional modeling studies on microbial community competition have shown that *G. sulfurreducens *is better adapted to acetate-rich environments, whereas *R. ferrireducens *thrives in nutrient-depleted environments (K. Zhuang, personal communication). Understanding these survival strategies is crucial for modeling complex microbial communities.

#### Central metabolism

*R. ferrireducens *possesses a full tricarboxylic acids (TCA) cycle and pentose phosphate pathway. Oxaloacetate is likely replenished by the combined action of PEP carboxylase (Rfer_1714) and pyruvate phosphate dikinase (Rfer_0088). Genes coding for the enzymes of the glyoxylate cycle and a glyoxylate oxidase (Rfer_0480-81) are present, which allow this organism to utilize glycolate as the sole electron and carbon source (data not shown).

The versatility in donor utilization is reflected by the existence of several pathways and their associated enzymes by which the key intermediate pyruvate can be produced. These include the enzymes of the glycolytic pathway, L-lactate dehydrogenase (cytochrome) [Rfer_2351, EC 1.1.2.3], and the pyruvate-oxidoreductase (POR) complex. Likewise, the genome provides multiple alternatives for generating acetyl-CoA: pyruvate dehydrogenase, aldehyde dehydrogenase/acetaldehyde dehydrogenase, acetate-CoA ligase and acetate kinase/phosphate acetyltransferase. Notably, of these enzymes only the pyruvate dehydrogenase and acetate kinase/phosphate acetyltransferase are shared by *G. sulfurreducens*.

No fermentative growth with sugars was observed in *R. ferrireducens*, which contrasts with other *Rhodoferax *species [2,10,11]. Fermentative growth on glucose was simulated with the *in silico *model and the result confirmed the experimental observation. Detailed analysis of the metabolic network suggested that the lack of fermentative growth of *R. ferrireducens *with glucose is likely due to its inability to recycle reduced NADH generated from glycolysis for redox balance without an electron acceptor. Compared to the *E. coli *metabolic model, the *R. ferrireducens *model lacks several reactions, including the reversible lactate dehydrogenase (LdhA), the pyruvate formate lyase (PflA), and the acetaldehyde CoA dehydrogenase/alcohol dehydrogenase (AdhE). These reactions are important to the *E. coli *fermentative growth that produces acetate, ethanol, lactate, and formate to allow the balance of the reducing equivalents generated during glycolysis [23]. Simulations with the *R. ferrireducens *model predicted that introducing any one of these enzymes into *R. ferrireducens *should support the fermentation of glucose (data not shown).

*R. ferrireducens *differs from most other acetate-oxidizing Fe(III) reducers in its ability to completely oxidize sugars to carbon dioxide with electron transfer to Fe(III) and electrodes [1]. The genome contains genes coding for the Entner-Doudoroff glycolytic pathway, typical of *Pseudomonads *and *Comamonas*. Sugars are likely to be imported into the bacterial cells by a homolog of a general hexose phosphotransferase system (Rfer_0601-03). In addition, several ABC transporters might be related to sugar transport. For instance, Rfer_0952-55 have weak similarity to ABC-type sugar transporters and are surrounded by genes related to carbohydrate metabolism (see Additional file 2). A larger cluster comprising CDSs Rfer_1094 to Rfer_1113 contains at least two putative sugar ABC transporters as well as other genes involved in sugar metabolism. *R. ferrireducens *is able to oxidize other sugars such as fructose, sucrose and mannose, but not lactose (not shown). The presence of two putative betaglucosidases in the genome (Rfer_1102 and Rfer_1111) suggested that *R. ferrireducens *might also be able to metabolize cellobiose, a fact confirmed in subsequent growth studies (see Additional file 3). Cellobiose degradation is of biotechnological interest because of the potential of turning common cellulosic waste products into energy. The *in silico R. ferrireducens *model includes the pathway for cellobiose degradation and predicts growth on this substrate.

#### Fumarate dismutation

**Figure 5:**
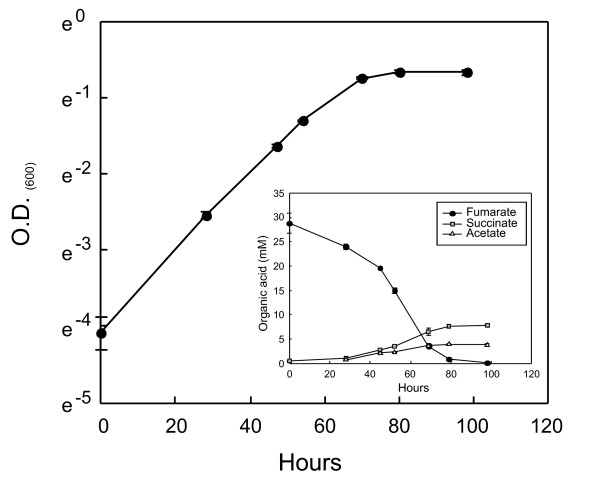
**Fumarate dismutation**. A) Growth on fumarate as the sole substrate. Inset: production of succinate and acetate as a result of fumarate consumption. Each point is the average of triplicate cultures with standard deviations.

New experimental evidence indicates that *R. ferrireducens *is able to grow with fumarate as the sole electron donor, electron acceptor and carbon source (Figure 5). This type of metabolism is known as fumarate dismutation and has been previously described in *Desulfovibrio *sp. [24], and the main products in *R. ferrireducens *are acetate and succinate. The *R. ferrireducens *genome contains genes whose products account for this metabolism, including a fumarate transporter DcuB (Rfer_3576) with 79% identity to the characterized homolog from *Wolinella succinogenes *[25] and a putative malate dehydrogenase (oxaloacetate-decarboxylating) (NADP^+^) (malic enzyme) with 89% identity to its homolog from *P. aeruginosa *(Rfer_0093). In fumarate dismutation simulations, fumarate is reduced to succinate accepting electrons from reducing equivalents that are generated in: 1) from fumarate through malic enzyme to acetyl-CoA, or 2) from fumarate to acetyl-CoA and the complete oxidation of acetyl-CoA through the TCA cycle. The first route is more efficient in generating ATP, but the *in silico *model predicted that the second route is also active in simulations constrained with experimental data, probably due to the high activities of the TCA cycle enzymes.

#### Citrate utilization

Citrate tested negative as an electron donor in the initial description of *Rhodoferax ferrireducens *[2]. However, other members of the *Rhodoferax *genus are able to utilize citrate [10], and examination of the genome suggests that it might be the case for *R. ferrireducens *as well. Rfer_2412 (CitT) is 44% identical to a putative citrate transporter from *E. coli *CFT073 [26] and is located in a cluster of genes also associated with citrate metabolism. Further experimental evaluation confirmed that *R. ferrireducens *could utilize citrate as electron donor and carbon source with Fe(III) or nitrate as the electron acceptor (see Additional file 4). Even though other *Betaproteobacteria *can also use citrate [27], BLAST-based analyses revealed that these *R. ferrireducens *citrate-related proteins are more closely related to *Gammaproteobacteria *and *Alphaproteobacteria *homologs (see Additional file 5). An exception is isocitrate dehydrogenase (Rfer_2411), for which there is a lineage-specific gene duplication in the *R. ferrireducens *genome (Rfer_2380, 97% identity) that resembles homologs of the *Betaproteobacteria*. Rfer_3489, annotated as a CitB, a citrate-utilization protein, might also be involved in this metabolism.

**Figure 6:**
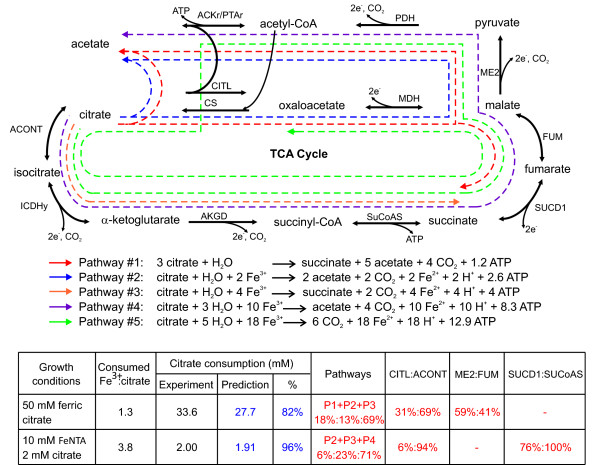
**Growth of *R. ferrireducens *with citrate as electron donor and Fe(III) as electron acceptor**. A) Possible pathways of citrate utilization: by-products differ depending on the availability of electron acceptor Fe(III). The different stoichiometries are summarized. B) *R. ferrireducens *is predicted to utilize a combination of different pathways during growth with limited Fe(III) availability. The numbers in red represent predicted fluxes.

The *in silico *model predicted that *R. ferrireducens *could completely oxidize citrate with Fe(III) as electron acceptor (Figure 6A, Pathway P5). However, this requires a very high Fe(III) flux, 18-fold higher than that of citrate. When culturing *R. ferrireducens *it was difficult to provide enough Fe(III) for complete oxidation of the citrate added. Therefore, the experimental growth of *R. ferrireducens *was under electron acceptor limiting conditions, which resulted in incomplete oxidation of citrate and production of acetate and succinate. The *in silico *model suggested four other pathways of citrate oxidation that could also produce acetate and/or succinate under acceptor limiting conditions. These pathways have different Fe(III):citrate stoichiometry and generate different amounts of ATP (Figure 6A, Pathways P1-P4). Citrate lyase (CITL), a key enzyme in citrate utilization, is active in pathways P1 and P2. The reversible malate dehydrogenase (MDH) can proceed in both directions: reductive in pathways P1 and P2, and oxidative in pathway P5. Succinate dehydrogenase can act in both directions, too: it is reductive in pathway P1 but oxidative in pathways P4 and P5. In order to figure out which of these pathways are the most likely to be active *in vivo*, citrate consumption was simulated *in silico *with data from two independent growth experiments applied as constraints (Figure 6B). The model predicted that three pathways were active. When the Fe(III):citrate ratio was low, *R. ferrireducens *utilized pathways P1, P2, and P3; which have low requirement of Fe(III) availability. When more Fe(III) was available, *R. ferrireducens *shifted towards pathway P4, which uses electron acceptors with a higher efficiency and more ATP is generated per citrate consumed (Figure 6B). The integration of experimental data and computational modeling in this case greatly improved our understanding of citrate metabolism, and can be readily applied to understanding other aspects of cellular physiology.

#### Degradation of aromatic compounds

**Figure 7:**
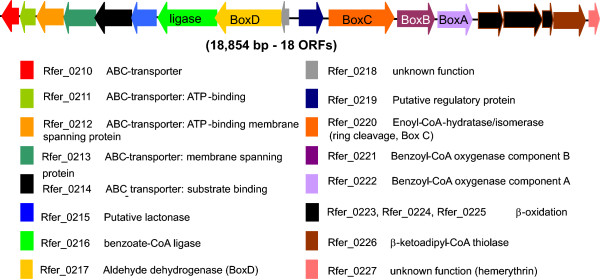
**Cluster of genes involved in the aerobic "hybrid" benzoate degradation pathway**.

The genome of *R. ferrireducens *harbors genes for benzoate degradation that are likely to be active under both aerobic and anaerobic conditions. Interestingly, unusual genes and enzymes are involved in both cases. Recently, a novel pathway for aerobic benzoate catabolism was characterized in *Azoarcus evansii *[28,29]. The unorthodox pathway combines features from the classical aerobic and anaerobic pathways of aromatic catabolism. In addition to an oxygen-dependent hydroxylation step similar to the aerobic route, it has the following features which are characteristic of the anaerobic pathways [30]; (1) the substrate is activated by co-enzyme A (CoA) formation, (2) the intermediates are processed as CoA thioesters, (3) ring cleavage is non-oxygenolytic, and (4) a β-oxidation-like reaction sequence is involved in the last steps of the pathway. In *R. ferrireducens *the genes of the new catabolic route are clustered and their products are most similar to orthologs from *Polaromonas naphtalenivorans*, one of the closest relatives of *R. ferrireducens *(Figure 7). Interestingly, *R. ferrireducens *also possesses genes coding for protocatechuate-4,5-dioxygenase (Rfer_0330, Rfer_0331), which catalyzes the oxygen-dependent ring cleavage of protocatechuate, an intermediate in aerobic benzoate degradation pathway.

In the absence of oxygen, the first step in benzoate catabolism is the production of benzoyl-CoA. The fact that there is only one gene for benzoate-CoA ligase in the *R. ferrireducens *genome (Rfer_0216) suggests that benzoyl-CoA formation is catalyzed by the same enzyme under both aerobic and anaerobic conditions, as observed in the denitrifying organism *Thauera aromatica *[31]. In facultative anaerobes, benzoyl-CoA is reductively dearomatized by an ATP-dependent benzoyl-CoA reductase, but the genes coding for this enzyme could not be found in the *R. ferrireducens *genome. It has been recently postulated that benzoyl-CoA reduction in obligate anaerobes like *Geobacter metallireducens *is mediated by a novel ATP-independent enzyme complex encoded by the genes *bamB-I *[32,33]. The genome of *R. ferrireducens *contains several genes whose products are moderately similar (based on percent similarity at protein-level) to some *bam *genes of *G. metallireducens *(see Additional file 6), but they do not appear to form a cluster. The possibility of *R. ferrireducens *having a novel system for benzoyl-CoA reduction cannot be excluded. The full elucidation of these pathways warrants further study. The *R. ferrireducens *model contains a pathway for benzoate degradation, and predicts the growth of *R. ferrireducens *on benzoate with Fe(III) as an electron acceptor.

The genome of *R. ferrireducens *also contains a putative pathway for the anaerobic catabolism of phenylalanine that includes a transaminase (Rfer_2174), a phenylpyruvate decarboxylase (Rfer_0518) and phenylacetaldehyde dehydrogenase (Rfer_0598). The end product of this pathway, phenylacetate, could be converted into benzoyl-CoA by the successive action of putative phenylacetate-CoA ligase (Rfer_3536) [34], phenylacetyl-CoA: acceptor oxidoreductase (Rfer_3093, Rfer_3094) and phenylglyoxylate:acceptor oxidoreductase (Rfer_2184-87) [35].

The complete oxidation of benzoate requires an elevated Fe(III):substrate ratio. This suggests that benzoate or other aromatic compounds could be a good feedstock for *R. ferrireducens *based microbial fuel cells to generate electricity. Thus, using aromatic waste stream for *R. ferrireducens *based microbial fuel cells could be an attractive idea that can achieve both bioremediation of aromatic compound contaminants and generation of electricity.

#### Analysis of substrate efficiency

**Figure 8:**
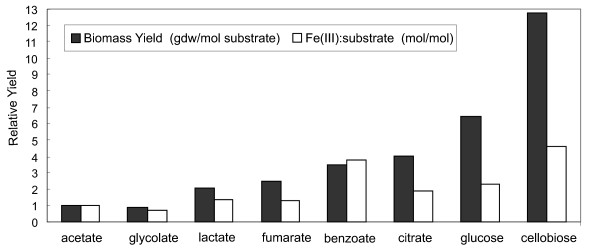
**Analysis of substrate efficiency**. Eight representative electron donors (acetate, glycolate, lactate, fumarate, benzoate, citrate, glucose, and cellobiose) were used in simulations of *R. ferrireducens *growth under donor-limiting conditions with Fe(III) as electron acceptor. The biomass yield to electron donor (gdw/mol substrate) and the Fe(III):substrate ratio from simulation results of *R. ferrireducens *growth on acetate were set at 1. The biomass yields to other donors and the ratios of Fe(III) to other electron donors are expressed in comparison to that of acetate.

The ability to predict growth yields on various substrates can be helpful for understanding the growth of microorganisms in various environments as well as for practical applications. *R. ferrireducens*, growth on eight representative electron donors (acetate, glycolate, lactate, fumarate, benzoate, citrate, glucose, and cellobiose) was simulated under electron donor-limiting conditions with Fe(III) as electron acceptor (Figure 8). Among the C6 compounds, glucose was predicted to yield more biomass than equivalent molar concentrations of citrate or benzoate. The simulations with different complex electron donors will allow a fast and easy analysis of substrate efficiency for bioremediation and electricity generation applications.

#### Photosynthesis and autotrophic growth

Although other members of the *Rhodoferax *genus are capable of autotrophic growth [10,11], this is not the case for *R. ferrireducens *[2]. No evidence of photosystems I or II was found in the genome. However, incomplete pathways associated with CO_2 _fixation are present. For instance, there is a gene coding for the large subunit of Ribulose-1,5-Bisphosphate Carboxylase/Oxygenase (RuBisCO) (Rfer_1391), but not the small subunit. Some genes encoding enzymes for the Calvin-Benson-Bassham cycle are present as well, but genes for phosphoketolase and seduheptulose-bisphosphatase are missing. The reductive carboxylate cycle, present in many photosynthetic bacteria, is also incomplete as a gene for the key enzyme ATP citrate synthase, is missing.

#### Cytochrome content

Well-studied Fe(III)-reducing microorganisms such as *Shewanella *and *Geobacter *species have abundant *c*-type cytochromes that are essential for extracellular electron transfer [36,37]. *R. ferrireducens *also has an abundance of *c*-type cytochrome genes (see Additional file 7). Based on matches to Prosite and hidden Markov Model (HMM) profiles, the *R. ferrireducens *genome possess 69 putative *c*-type cytochromes, of which 45 have matched above the high confidence scores to the Prosite profiles for the Cytochrome *c *family (PS51007) and multiheme cytochrome *c *family profiles (PS51008). Of the *R. ferrireducens *putative *c*-type cytochrome complement, approximately 45% (31/69) possess a homolog to a *c*-type cytochrome indentified in a previously sequenced [36]*Geobacter *spp. genome and the majority appears to reside in the periplasm or outer membrane based on the presence of predicted signal peptides. One *c*-type cytochrome gene in *R. ferrireducens*, Rfer_0244, is a homolog of OmcE, an outer-membrane *c*-type cytochrome that is essential for Fe(III) oxide reduction in *G. sulfurreducens *[38]. However, there are no homologs to several other cytochromes shown to be important in *G. sulfurreducens *Fe(III) oxide reduction (and electricity generation) including OmcB, OmcC, OmcF, OmcS, OmcT and OmcZ [38-40]. These results suggest that *c*-type cytochrome complements can vary in Fe(III) oxide reducing (and electricity producing) prokaryotes. However, whether or not a subset of *c*-type cytochromes essential to Fe(III) oxide reduction (or electricity generation) exists within the set shared between *Rhodoferax *and other Fe(III) oxide reducing prokaryotes remains to be determined. This may not be surprising as there is poor conservation of cytochromes even among *Geobacter *species (J. Butler, personal communication).

#### Nitrogen metabolism

**Figure 9:**
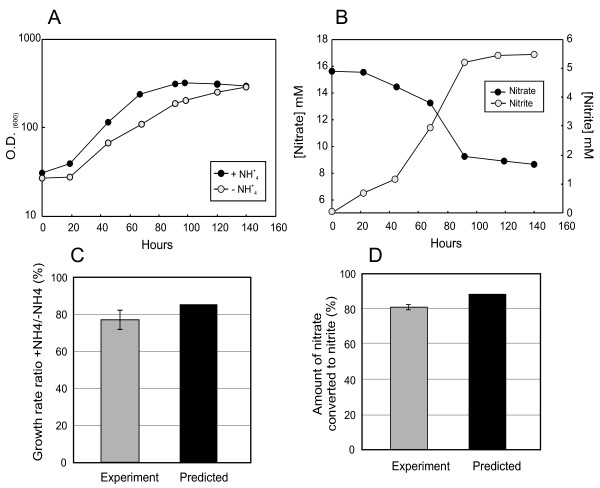
**Growth on nitrate as electron acceptor and the sole N source**. A) Growth on 0.1% glucose and 15 mM nitrate. Ammonia (0.25 g/L) was either added or omitted from the medium. B) Production of nitrite. The medium contained 0.1% lactate and 20 mM nitrate, ammonia was omitted. C) Experimental *vs*. predicted growth rate ratios with and without added ammonia. D) Experimental *vs*. predicted ratio of nitrate converted to nitrite.

The genome of *R. ferrireducens *contains a respiratory nitrate reductase complex (Rfer_2792-95), and nitrate was previously reported to serve as an electron acceptor supporting growth [2]. Further investigation revealed that nitrate is reduced to nitrite (Figure 9A). Although genes coding for nitric oxide and nitrous oxide reductases are present (Rfer_1886 and Rfer_3199, respectively), *R. ferrireducens *is not a denitrifier, probably due to the absence of a NO-forming nitrite reductase. Approximately 80% of the nitrate was converted to nitrite (Figure 9B), thus failing the criteria that at least 80% of the nitrogen ought to be recovered as gas for an organism to be considered a denitrifier [41].

*R. ferrireducens *also contains assimilatory forms of nitrate reductase (Rfer_2559) and nitrite reductase NAD(P)H (Rfer_2557-2558) that allow growth on nitrate without the addition of ammonia, although the growth rate was higher in the ammonia-amended cultures (Figure 9A).

The *R. ferrireducens *model contains reactions that account for nitrate utilization as electron acceptor and nitrogen source. It predicts a 20% faster growth rate if ammonium is added to the medium, in agreement with actual experimental results (Figure 9A and 9C). The model also accurately predicted the proportion of nitrate converted to nitrite, *ca*. 80% (Figure 9B and 9D). The rest is likely converted to ammonium, of which 60% is predicted to be assimilated into biomass, probably via glutamine synthetase.

#### Metalloid and metal resistance

The *R. ferrireducens *genome contains a cluster of genes related to arsenic metabolism (see Additional file 8). The gene cluster Rfer_3663-3665 encode respectively: a putative arsenite efflux pump; an arsenite-activated ATPase and a arsenate reductase which are likely to be involved in removing arsenic from the cell. However, tolerance to arsenic has yet to be evaluated.

The genome contains two genes, Rfer_2447 and Rfer_2824, which have 30% and 27% identity respectively to the characterized chromate transporter homolog, ChrA, in *P. aeruginosa *[42]. ChrB (Rfer_2446), on the other hand, is 58% identical to the Cr(VI)-sensing regulator ChrB in *Ochrobactrum triciti*, an *Alphaproteobacteria *isolated from chromium-contaminated sludge and able to grow in the presence of high concentrations of chromium [43-45]. A chromate reductase (ChrR) homolog was not evident. The *R. ferrireducens *genome also encodes a heavy metal efflux pump CzcA (Rfer_0411), which might confer resistance to Cd, Zn and Co [46]. Other genes encoding proteins involved in metal resistance are four putative copper-translocating P-type ATPases (Rfer_0024, Rfer_0418, Rfer_1144 and Rfer_1927) and a periplasmic copper-binding protein (Rfer_3200).

#### Storage capabilities: Polyhydroxyalkanoates

Many organisms have the ability to trigger the production of carbon storage compounds under unfavorable conditions such as limited or inaccessible electron acceptors and/or lack of key nutrients (*e.g*., nitrogen or sulfate) [47]. Some of these polymers have attractive physical properties that make them relevant for industrial use, particularly in biodegradable materials [12]. Genome analysis indentified three genes well characterized for their involvement in PHA synthesis present in a putative operon in the *R. ferrireducens *genome: Acetoacetyl-CoA reductase (Rfer_2560), Acetyl-CoA acetyltransferase (Rfer_2561) and PHA synthase (Rfer_2562). Even though production of PHAs has been previously observed in *R. ferrireducens *[2], the *in silico *model does not account for this pathway. It is expected to be included once experimental data are available.

#### Response to environmental challenges

*R. ferrireducens *is a psychrotolerant organism that can withstand temperatures as low as 4°C, which might confer a competitive advantage in certain environments [2]. Notably, no genes coding for major cold shock proteins (Csp) (as identified by matches above trusted cutoffs to PF00313: cold-shock DNA-binding domain or TIGR02381: cold shock domain protein CspD) could be identified in the *R. ferrireducens *genome or other members of the *Comamonadaceae *whose genomes have been sequenced (*Polaromonas *spp. and *Acidovorax *sp. JS42). Other members of the *Betaproteobacteria *for which whole genome sequence is available (from the genera *Burkholderias, Azoarcus, Nitrobacter *and *Ralstonia*) possess these Csp homologs. This suggests that other cold shock proteins or other mechanisms for surviving cold shock events have yet to be identified in *R. ferrireducens*.

*R. ferrireducens *is a facultative organism and can utilize atmospheric oxygen as a terminal electron acceptor. The genome contains a cytochrome *c*-oxidase and also several genes coding for enzymes related to the oxidative stress response, such as superoxide dismutase (Rfer_3151) and several alkylhydroxyperoxidases (see Additional file 9).

Examination of the *R. ferrireducens *genome revealed that this organism has potential to respond to a wide variety of stimuli, with over 30 genes coding for putative sensor histidine kinases, 23 methyl-accepting chemotaxis proteins (MCPs) and 32 DNA-binding response regulator elements. The genes coding for sensor proteins and response regulators (possibly two-component systems) are often found in pairs, and at least 47 such pairs were found in the *R. ferrireducens *genome (see Additional file 10). Homologs of CheA, CheW, CheV and CheY are also present, though the chemotactic behavior of *R. ferrireducens *is largely unknown.

*R. ferrireducens *is motile by means of one polar flagellum [2]. Genes coding for flagellin-like proteins have been identified (Rfer_0630 and Rfer_0631) that cluster with genes coding for a flagellar hook-associated protein (Rfer_0632) and for flagellin-specific chaperons FliS and FliT [48]. At least 35 genes are directly (components of the flagellar apparatus) or indirectly (specific chaperones, regulators) related to flagellar motility and are grouped in two clusters in the genome.

#### CRISPR sequences and immunity to phage attack

A 5.7 kb array of clustered regularly interspaced short palindromic repeats (CRISPR) was identified in the chromosome of *R. ferrireducens*, using the CRISPR Recognition Tool [49]. It has been previously shown in a different bacterial system that new CRISPR spacer sequences derived from phage genomic DNA are added after viral challenge [50]. The presence of these sequences in the host genome was shown to confer phage-resistance, in association with the *cas *genes. Thus, the CRISPR locus in the chromosome of *R. ferrireducens *may protect against phage attack. In addition, there is a 1.5 kb CRISPR array in the plasmid, encoding 24 spacer sequences of 32 bp. The CRISPRs in the chromosome and plasmid are 37 bp and 32 bp respectively, and these two loci are associated with separate sets of *cas *genes. Thus, the plasmid-encoded CRISPR appears independent of the chromosome-encoded CRISPR. Spacer sequences in both loci have no significant matches to entries in the NCBI databases (except to themselves). The results suggest that a significant cache of horizontally transferrable genetic elements which the *R. ferrireducens *chromosome and plasmid have encountered previously have yet to be sequenced and identified. Alternatively, if the horizontal acquisition of these elements occurred a long time ago in terms of *R. ferrireducens *evolution and selective pressure to maintain the original sequence is low, these spacer regions may have been ameliorated to such a degree that homology to the original sequence is no longer detectable.

## Conclusion

**Figure 10:**
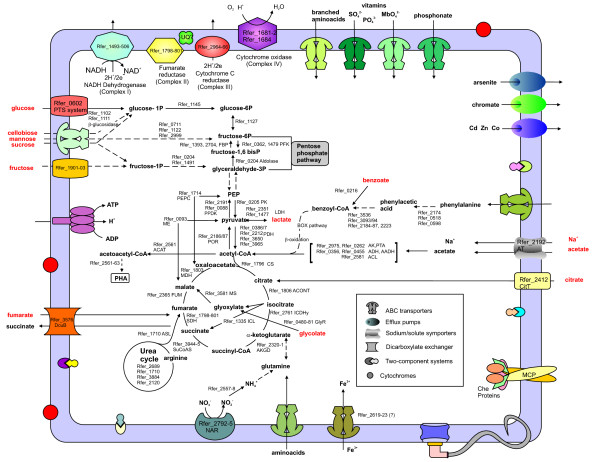
**Schematic representation of relevant metabolic and physiological features**.

This study demonstrates how genome-scale metabolic modeling, coupled with enhanced genome annotation and laboratory studies, can accelerate the study of the physiology of environmentally relevant, but understudied microorganisms. In relatively short order the understanding of metabolism of *R. ferrireducens *has advanced from a description of growth characteristics [1,2] to a detailed understanding of metabolism (Figure 10) and the ability to predict the growth and metabolism of *R. ferrireducens *under a diversity of environmental conditions. The genome-scale metabolic model of *R. ferrireducens *is expected to be a useful tool for studying the role of this microorganism in soils and sediments and its interactions with other microorganisms, such as *Geobacter *species, that share some physiological characteristics. Furthermore, the genome-scale metabolic model of *R. ferrireducens *is likely to be a powerful tool for optimizing potential applications of *R. ferrireducens *such as the bioremediation of contaminants, the production of polymers, and the conversion of organic compounds to electricity.

## Methods

### Bacterial strains and culturing conditions

*Rhodoferax ferrireducens *strain DMS 15236 (ATCC BAA-621) [2] was obtained from our laboratory collection. A defined freshwater medium was used to culture and propagate this bacterium [51]. Electron donors were added as follows: acetate 10 mM, lactate 0.1%, glycolate 10 mM, citrate 10 mM, glucose 0.1%, mannose 0.1%, sucrose 0.1%, cellobiose 0.1%. Electron acceptors were added as follows: fumarate 30 mM, ferric citrate 56 mM, Fe(III)NTA 5 mM, nitrate 20 mM.

### Determination of growth yields

*Rhodoferax ferrireducens *cells were collected at stationary phase, centrifuged at 4000 RPM for 15 minutes at 4°C and washed with isotonic buffer [52]. Any remaining liquid was carefully removed with a pipette and the pellets were flash-frozen for storage at -80°C. Cell pellets were subsequently resuspended in 5% SDS and boiled for 10 minutes. Protein concentrations were determined with the bicinchoninic acid (BCA) method using bovine serum albumin as standard [53]. Dry weight was calculated assuming that protein accounts for 55% of the cell mass according to the biomass equation derived from *E. coli *model [21]. Biomass yield from published acetate and Fe(III) data [2] was calculated using the value from *E. coli *(10^-12 ^g/cell).

### Analytical techniques

Growth of fumarate cultures was assessed by measuring optical density at 600 nm with a Genesys 2 spectrophotometer (Spectronic Instruments, Rochester, NY). The organic acid content of the culture medium was determined by high-pressure liquid chromatography (HPLC) using an LC-10AT high-pressure liquid chromatograph (Shimadzu, Kyoto, Japan) equipped with an Aminex HPX-87H column (300 by 7.8 mm; Bio-Rad, Hercules, CA). Organic acids were eluted in 8 mM H_2_SO_4 _and quantitated with an SPD-10VP UV detector (Shimadzu, Kyoto, Japan) set at 215 nm. Nitrate and nitrite concentrations were determined with a Dionex DX-100 ion chromatograph. Fe(II) concentrations were determined with the ferrozine assay as previously described [54].

### Annotation and comparative genomic analyses

The *R. ferrireducens *genome was originally sequenced and annotated at the DOE Joint Genome Institute (JGI)  and assigned GenBank accession number CP000267 and CP000268 for the chromosome and plasmid, respectively. In order to enhance our ability to perform comparative genomic analyses and to generate a more accurate *in silico *constraints based model, further manual curation of the original genome assembly (chromosome and plasmid) was completed at the J. Craig Venter Institute (JCVI) as indicated in the following description. Results of the manual curation (as well as the original annotation from the JGI) are available at the Comprehensive Microbial Resource  maintained by the JCVI. An initial set of CDSs that likely encode proteins was identified using GLIMMER 3 and those shorter than 90 base pairs (bp) as well as some of those with overlaps eliminated. There are 4451 CDSs that have been predicted on the main chromosome and 319 on the plasmid for a total of 4770 CDSs in the genome. 4333 CDSs from the JCVI annotation mapped to JGI annotation in GenBank. For clarity and ease of access to data, locus tags as assigned by GenBank are used to indicate CDSs referred to specifically in the work presented here unless otherwise indicated. For clarity and ease of access to data, locus tags as assigned by GenBank are used to indicate CDSs referred to specifically in the work presented here unless otherwise indicated.

CDSs were searched against a non-redundant protein database as previously described [55]. Frameshifts and point mutations were detected and corrected where appropriate. Remaining frameshifts and point mutations are considered authentic and corresponding regions were annotated as 'authentic frameshift' or 'authentic point mutation', respectively. The CDS prediction and gene family identifications were completed as follows. Two sets of HMMs were used to determine CDS membership in families and superfamilies. These included 721 HMMs from Pfam v22.0 and 631 HMMs from the TIGR ortholog resource. TMHMM [109] was used to identify membrane-spanning domains (MSD) in proteins. Manual annotation, included adjustment of start sites, gene names and assignment to putative functional role categories.

Criteria for the identification of cytochrome genes used the Perl script "ps_scan.pl" and 3 Prosite profiles: PS51007 cytochrome c family profile, PS51008 multiheme cytochrome c family profile, and PS51009 cytochrome c class II profile. A gene scoring a low confidence cutoff (level = -1) was qualified as "putative" compared to a gene scoring above the reliable cutoff (level = 0). Additional HMM evidence and matches to a database of experimentally characterized genes maintained in house sometimes provided greater specificity to the corresponding gene name.

All predicted proteins from the *R. ferrireducens *genome were compared using bidirectional BLASTP to those from all other completed Proteobacterial genomes. Significant BLAST matches were scored for each CDS using cutoffs of 10^-5 ^for the *P *value, and 70% for the length of the alignment compared to both query and database proteins. CDSs with a top match to another *R. ferrireducens *protein rather than to a protein in another species were considered candidates for which recent lineage-specific duplication events have occurred.

### Metabolic network reconstruction

The *R. ferrireducens *metabolic network was reconstructed in SimPheny (Genomatica, Inc., CA) by modifying previously published procedures [56]. The annotated genes of the *R. ferrireducens *genome as well as genes from several high-quality genome-scale metabolic models, including previously published *Escherichia coli *[56], *Geobacter sulfurreducens *[22], and *Bacillus subtilis *[57] models, were utilized for BLASTP sequence similarity search to generate a draft network as a starting point for model reconstruction. Among the base models used, *E. coli *was the phylogenetically closest to *R. ferrireducens *and provided about half of all reactions in the draft model, which captured significant portions of central metabolism, biosynthetic pathways for amino acids, nucleotides, and lipids. The reactions and their gene associations in the draft model of *R. ferrireducens *were evaluated manually based on gene annotations, published biochemical and physiological information, and external references as previously described [58] The remaining genes were also reviewed for inclusion in the reconstructed network. During this process, reactions and pathways not in the base models were identified, validated, and added into the *R. ferrireducens *model. A biomass demand reaction based on biomass compositions of the published *E. coli *model [56] was used in the *R. ferrireducens *model (See Additional file 1 for details). Exchange reactions for all extracellular compounds were added. The resulting reconstructed network was then subjected to the gap filling process where gaps were identified and filled manually through simulations to allow biomass formation under physiological growth conditions that include growth with Fe(III)-NTA, nitrate and oxygen as electron acceptors and acetate, propionate, lactate, pyruvate, malate, succinate, benzoate and glucose as electron donors [1,2].

### Estimation of energy parameters of the metabolic model

Energy parameters of the metabolic model including GAM, NGAM and proton translocation stoichiometry were estimated as follows. The GAM requirement for the *R. ferrireducens *model was assumed to be the same as the *E. coli *model at 59.81 mmol ATP/gdw h [21] based on their similar biomass compositions. The NGAM requirement and the proton translocation stoichiometry (H^+^/2e^-^, number of protons per pair of electrons) for NADH dehydrogenase and cytochrome reductase were estimated by iterating for optimization between *in silico*-predicted and experimentally determined growth yields. Observed experimental growth rates were all less than 0.04 h^-1^, so all *in silico *simulations were constrained with a growth rate less than 0.04 h^-1^. The experimental data used to determine the energetic parameters were obtained from three independent experiments where *R. ferrireducens *was cultivated in batch under the following conditions: a) acetate and fumarate, b) citrate and Fe(III), and c) acetate and Fe(III) [2].

### *In silico *analysis of metabolism

The metabolic capabilities of the *R. ferrireducens *network were calculated using flux balance analysis and linear optimization [56] in SimPheny. Biomass synthesis was selected as the objective function to be maximized in growth simulations, and ATP consumption was selected as the objective function to be maximized in energy requirement simulations. The simulations resulted in flux values in units of mmol/gdw h. The following external metabolites were allowed to freely enter and leave the network for simulations of anaerobic growth on minimal media: CO_2_, H^+^, H_2_O, K^+^, Mg^2+^, NH_4_^+^, phosphate, and sulfate. A minimal amount of oxygen was allowed for biomass component requirements. The electron donors or acceptors tested were allowed a maximum uptake rate into the network of 5 mmol/gdw h or as specified in the results. All other external metabolites were only allowed to leave the system.

## List of Abbreviations

AADH: acetaldehyde dehydrogenase; ACAT: acetyl-CoA acetyltransferase; ACKr: acetate kinase; ACL: acetate-CoA ligase; ACONT: aconitase; ADH: aldehyde dehydrogenase; AKGD: 2-oxoglutarate dehydrogenase; ASL: argininosuccinate lyase; AT: acetate transporter; CITL: citrate lyase; CS: citrate synthase; FBP: fructose-1,6-bisphosphate phosphatase; FUM: fumarase; gdw: grams of dry weight; GlyR: glycolate reductase; ICDHy: isocitrate dehydrogenase; ICL: isocitrate lyase; IDH: isocitrate dehydrogenase; LDH: lactate dehydrogenase; MDH: malate dehydrogenase; ME2: malic enzyme (NADP); MS: malate synthase; NAR: nitrate reductase; NTA: nitriloacetic acid; PDH: pyruvate dehydrogenase; PEPC: phosphoenolpyruvate carboxylase; PFK: phosphofructokinase; PK: pyruvate kinase; POR: pyruvate oxidoreductase complex; PPDK: pyruvate phosphate dikinase; PTAr: phosphotransacetylase; SCS: succinyl-CoA synthetase; SDH: succinate dehydrogenase; SUCOAS: succinyl-CoA synthetase; SUCD1: succinate dehydrogenase.

## Authors' contributions

CR carried out all the growth and physiology experiments, analyzed the data, drafted part of the manuscript and coordinated the project. JS and OB developed the genome-scale metabolic model. JS completed the *in silico *simulations, analyzed the experimental data and drafted part of the manuscript. KZ and KM developed the computational algorithm for analyzing the energetic parameters. RD performed comparative genome analyses including evaluations of CRISPRs, conceived and wrote the program for BLAST Curves, and aided in data interpretation. WI performed the analysis of the pathways for the degradation of aromatic compounds. SS, HH, and SK performed manual annotation of the genome. SD performed manual annotation of the genome, completed comparisons between original JGI genome annotation and the improved version used for modeling and produced all files needed as input for modeling. BAM coordinated and contributed to all genome annotation and comparative genomic analyses, interpreted data, conceived ideas and contributed with text for the manuscript. JS, KM, CHS and DRL conceived the study and revised the manuscript. All authors read and approved the final manuscript.

## Supplementary Material

Additional file 1 **List of reactions, genes and metabolites included in the *R. ferrireducens in silico *model**. This spreadsheet includes a detailed list of all the reactions included in the model: intracelullar reactions, exchange reactions and biomass equation. Click here for file

Additional file 2 **Carbohydrate metabolism in *R. ferrireducens *genome**. Spreadsheet listing putative genes related to the transport and metabolism of mannose, fructose and cellobiose. Click here for file

Additional file 3 ***R. ferrireducens *can use cellobiose as electron donor and carbon source**. Growth curve of *R. ferrireducens *on 0.1% cellobiose and 0.5 mM Fe(III)NTA. Click here for file

Additional file 4 **Citrate metabolism in *R. ferrireducens***. Spreadsheet listing putative genes involved in the transport and metabolism of citrate. Click here for file

Additional file 5 **Citrate as the sole electron donor and carbon source**. Growth curve of *R. ferrireducens *on 56 mM ferric citrate. Click here for file

Additional file 6 **Putative genes involved in aromatic catabolism in *R. ferrireducens***. Table listing genes in the aerobic "hybrid" and the putative anaerobic pathways of benzoate and other aromatic compounds degradation in *R. ferrireducens*. Click here for file

Additional file 7 **Putative cytochromes *c *in the *R. ferrireducens *genome.** Click here for file

Additional file 8 **Putative genes involved in metal resistance in the *R. ferrireducens *genome**. Spreadsheet listing genes involved in the resistance to arsenite, chromate, copper and heavy metal efflux pumps. Click here for file

Additional file 9 **Chemotaxis proteins (Che) and Two-Component systems**. Spreadsheet listing genes coding for Che proteins, putative sensor histidine kinases, response regulators and methyl-accepting chemotaxis proteins (MCPs). Click here for file

Additional file 10 **Oxygen metabolism**. Spreadsheet listing putative cytochrome *c *oxidases as well as genes involved in oxidative stress. Click here for file
