# Supplementary material for: Genome-scale comparison and constraint-based metabolic reconstruction of the facultative anaerobic Fe(III)-reducer Rhodoferax ferrireducens
Source: BMC Genomics. 2009 Sep 22;10:447. doi: 10.1186/1471-2164-10-447 (PMC2755013; doi:10.1186/1471-2164-10-447)

Additional file 3

Title: Growth of *R. ferrireducens* on cellobiose as electron donor and carbon source.

File format: PDF

Description: Growth curve on 0.1% cellobiose and 500 μM Fe(III)NTA as electron donor. Each point represents the average of triplicate cultures with standard deviations.

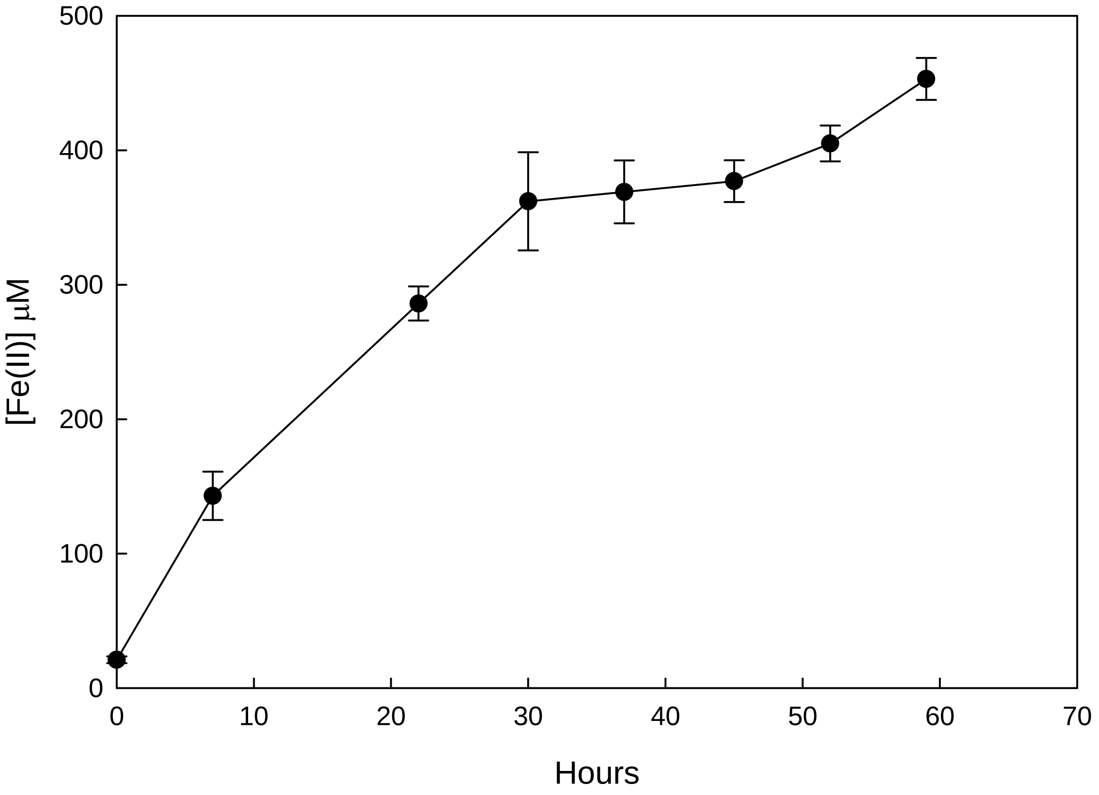

Supplement: Additional file 3 — R. ferrireducens can use cellobiose as electron donor and carbon source. Growth curve of R. ferrireducens on 0.1% cellobiose and 0.5 mM Fe(III)NTA. [file 1471-2164-10-447-S3.PDF]
