# Supplementary material for: Genome-scale comparison and constraint-based metabolic reconstruction of the facultative anaerobic Fe(III)-reducer Rhodoferax ferrireducens
Source: BMC Genomics. 2009 Sep 22;10:447. doi: 10.1186/1471-2164-10-447 (PMC2755013; doi:10.1186/1471-2164-10-447)

Additional file 4

Title: Growth of *R. ferrireducens* on ferric citrate.

File Format: PDF

Description: Growth curve on 56 mM ferric citrate. Each point represents the average of triplicate cultures with standard deviations.

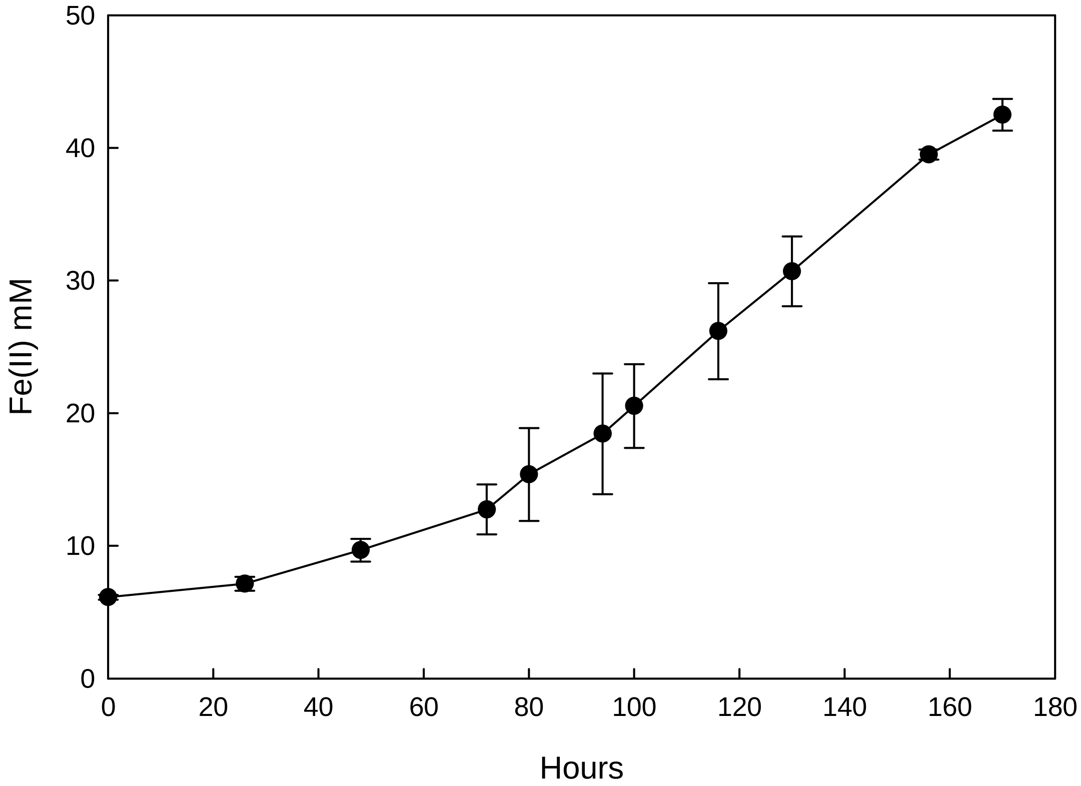

Supplement: Additional file 4 — Citrate metabolism in R. ferrireducens. Spreadsheet listing putative genes involved in the transport and metabolism of citrate. [file 1471-2164-10-447-S4.PDF]
