# Supplementary material for: Genome-scale comparison and constraint-based metabolic reconstruction of the facultative anaerobic Fe(III)-reducer Rhodoferax ferrireducens
Source: BMC Genomics. 2009 Sep 22;10:447. doi: 10.1186/1471-2164-10-447 (PMC2755013; doi:10.1186/1471-2164-10-447)
Supplement: Additional file 6 — Putative genes involved in aromatic catabolism in R. ferrireducens. Table listing genes in the aerobic "hybrid" and the putative anaerobic pathways of benzoate and other aromatic compounds degradation in R. ferrireducens. [file 1471-2164-10-447-S6.DOC]

**Additional file 6**

**Title: Genes involved in aromatics metabolism in *R. ferrireducens***

**File format: DOC**

**Description: List of genes involved in aromatics metabolism grouped by individual pathways.**

| **Gene ID** | **Putative function** |
| --- | --- |
| Aerobic or “hybrid” pathway of benzoate degradation | |
| Rfer_0210 | ABC-transporter, ATP binding |
| Rfer_0211 | ABC-transporter, ATP binding, membrane spanning |
| Rfer_0212 | ABC-transporter, ATP binding, membrane spanning |
| Rfer_0213 | ABC transporter, membrane spanning |
| Rfer_0214 | ABC transporter, substrate binding |
| Rfer_0215 | Lactonase |
| Rfer_0216 | Benzoate-CoA ligase |
| Rfer_0217 | 3,4-dehydroadipyl-CoA semialdehyde dehydrogenase (Box D) |
| Rfer_0218 | Unknown function |
| Rfer_0219 | Transcriptional regulator |
| Rfer_0220 | Non-oxygenolytic ring cleavage (Box C) |
| Rfer_0221 | Benzoyl-CoA oxygenase (Box B) |
| Rfer_0222 | Benzoyl-CoA oxygenase/reductase (BoxA) |
| Rfer_0223 | β-oxidation |
| Rfer_0224 | β-oxidation |
| Rfer_0225 | β-oxidation |
| Rfer_0226 | Thiolase |
| Rfer_0227 | Unknown function |
|  |  |
| Anaerobic benzoate degradation pathway | |
| Rfer_0216 | Benzoate-CoA ligase |
| Rfer_2850 | Aldehyde: ferridoxin oxidoreductase (BamB) |
| Rfer_2851 | Fe-S binding protein (BamC) |
| Rfer_1497 | NADH:ubiquinone oxidoreductase (BamG) |
| Rfer_1498 | NADH:ubiquinone oxidoreductase (BamH) |
| Rfer_1499 | Uncharacterized anaerobic dehydrogenase (BamI) |
|  |  |
| Phenylacetate degradation | |
| Rfer_3536 | Phenylacetate-CoA ligase |
| Rfer_3094 | Phenylacetyl-CoA:acceptor oxidoreductase |
| Rfer_3093 | Phenylacetyl-CoA:acceptor oxidoreductase |
| Rfer_2187 | Phenylglyoxylate :acceptor oxidoreductase |
| Rfer_2184 | Phenylglyoxylate :acceptor oxidoreductase subunit |
| Rfer_2186 | Phenylglyoxylate :acceptor oxidoreductase subunit |
| Rfer_2223 | Phenylglyoxylate :acceptor oxidoreductase subunit |
| Rfer_2185 | Phenylglyoxylate :acceptor oxidoreductase subunit |
|  |  |
| Phenylalanine degradation | |
| Rfer_2174 | Phenylalanine transaminase |
| Rfer_0518 | Phenylpyruvate decarboxylase |
| Rfer_0598 | Phenylacetaldehyde dehydrogenase |
|  |  |
